# A *de novo*‐assisted strategy to identify novel lncRNA‐encoded peptides in cerebrospinal fluid of demented subjects with or without amyloid positivity

**DOI:** 10.1002/alz70856_106893

**Published:** 2026-01-08

**Authors:** Satya Saxena, Yuanqing Ye, Viswanath Devanarayan, Larisa Reyderman, Kristin R Wildsmith, Pallavi Sachdev

**Affiliations:** ^1^ Eisai Inc., Nutley, NJ, USA

## Abstract

**Background:**

Long non‐coding RNAs (lncRNAs) play a significant role in the pathogenesis of Alzheimer's disease (AD). They modulate various cellular processes such as amyloid production, Tau hyperphosphorylation, neuroinflammation, and the impairment of mitochondrial and synaptic functions. Emerging studies have revealed that certain lncRNAs can encode small open reading frame‐derived peptides. However, the identification and understanding of the role of lncRNA‐encoded peptides in AD remain largely unexplored due to the inherent low abundance and small sizes of these peptides. Here, we leveraged the *de novo* peptide sequencing algorithm and a custom database to identify lncRNA‐encoded peptides in cerebrospinal fluid (CSF) of demented subjects

**Method:**

We developed an innovative strategy to identify lncRNA‐encoded peptides in biofluids. A custom database of hypothetical peptides was generated by six‐frame translation of all lncRNAs from LNCipedia (www.lncipedia.org) and integrated to human SwissProt protein entries. MS data (PRIDE archive PXD016278) from CSF of demented subjects with (*n* = 29) or without (*n* = 31) amyloid positivity were analyzed. Peptide raw peak area intensities were quantile normalized and log2 transformed to reduce technical variation and ensure distribution symmetry. Differentially expressed peptides were identified via analysis of covariance after adjusting for age and gender, with the significant criteria based on 20% false discovery rate (FDR).

**Result:**

The *de novo*‐assisted search of MS spectra identified 32,191 peptides at 0.1% global *peptide*‐level *FDR*. Mapping of *de novo* peptides to our custom database identified 99 lncRNA‐encoded peptides in CSF of demented subjects. 7/99 significantly (q<0.2; Cohens >0.8) altered lncRNA‐encoded peptides were linked to amyloid positivity. These peptides were translated from non‐coding regions of six lncRNA genes involved in the cellular processes relevant to AD, including tau protein aggregation and glutamatergic synapse plasticity.

**Conclusion:**

This is the first study identifying differentially regulated lncRNA‐encoded peptides in the CSF of Ab+ (AD) and Ab‐ (non‐AD) dementia. Further investigation of novel lncRNA‐derived peptides lights a new beacon to explore their promising applications in AD diagnosis, staging, and future treatment strategies.

